# Single-cell sequencing shows mosaic aneuploidy in most human embryos

**DOI:** 10.1172/JCI179134

**Published:** 2024-03-15

**Authors:** Sarah A. Robertson, Robert I. Richards

**Affiliations:** 1 The Robinson Research Institute and School of Biomedicine and; 2 School of Molecular Bioscience, University of Adelaide, Adelaide, South Australia, Australia.

## Abstract

Mammalian preimplantation embryos often contain chromosomal defects that arose in the first divisions after fertilization and affect a subpopulation of cells — an event known as mosaic aneuploidy. In this issue of the *JCI*, Chavli et al. report single-cell genomic sequencing data for rigorous evaluation of the incidence and degree of mosaic aneuploidy in healthy human in vitro fertilization (IVF) embryos. Remarkably, mosaic aneuploidy occurred in at least 80% of human blastocyst-stage embryos, with often less than 20% of cells showing defects. These findings confirm that mosaic aneuploidy is prevalent in human embryos, indicating that the process is a widespread event that rarely has clinical consequences. There are major implications for preimplantation genetic testing of aneuploidy (PGT-A), a test commonly used to screen and select IVF embryos for transfer. The application and benefit of this technology is controversial, and the findings provide more cause for caution on its use.

## Chromosomal errors are common in human embryos

In human genetics, everything that can go wrong probably sometimes does. What matters is the frequency at which errors occur, whether they have clinical consequences, and the efficiency of biological mechanisms to correct faults or remove defective cells. It is well known that a common feature of mammalian preimplantation embryos is the presence of aneuploidy, in which cells gain or lose entire chromosomes or chromosome fragments, or exhibit chromosomes with structural rearrangements. When every cell in the embryo has the same defect — due to meiotic errors during sperm or oocyte development — the effects are often reflected by low viability, implantation failure, late miscarriage, or stillbirth. Nevertheless, fetuses with abnormalities of chromosomes 13, 18, 21, X, and Y can be and are carried to term, with marked and variable clinical consequences (1).

Many human embryos exhibit a second type of chromosomal abnormality called mosaic aneuploidy. In this event, embryos contain a mixture of euploid cells and aneuploid cells containing errors presumed to occur after fertilization (2). Indeed, chromosome defects often appear to arise in the first mitotic divisions, in which special features of cell cleavage predispose to segregation errors (3–5). When only some of the cells in an embryo are affected, most often the defective cells are lost or outcompeted during early development and are not retained in the fetus (3, 6, 7). This tolerance of errors means there is a good chance a mosaic embryo will give rise to a healthy infant. Although, occasionally, aneuploid cells survive in cases of mosaicism to confer symptoms on an affected tissue, to a degree that depends on a variety of factors including the prevalence of mosaic cells and the chromosome involved (1).

Recent research has raised the question of whether chromosomal mosaicism is a regular or even normal occurrence in early embryo development (2). This issue is important because genetic screening has become commonplace in human reproductive medicine. A tool known as preimplantation genetic testing for aneuploidy (PGT-A) seeks to allow preferential selection of embryos with no sign of aneuploidy for transfer to initiate pregnancy. But given recent information on the implications of mosaic aneuploidy, as well as concerns about the precision and sensitivity of the PGT-A test and its associated risks and cost, whether testing is a useful and desirable component of routine in vitro fertilization (IVF) treatment is now a topic of hot debate (2, 8).

## Single-cell sequencing shows mosaic aneuploidy in most human embryos

In this issue of the *JCI*, Chavli, Klaasen, and colleagues (9) provide insight that profoundly affects the debate on the application and benefit of PGT-A. Their single-cell, whole-genome sequencing (sc-Karyo-Seq) analysis provides compelling evidence that more than 80% of high-quality human IVF embryos exhibit chromosomal defects in a mosaic distribution, often affecting only a small proportion of cells.

**Figure 1:**
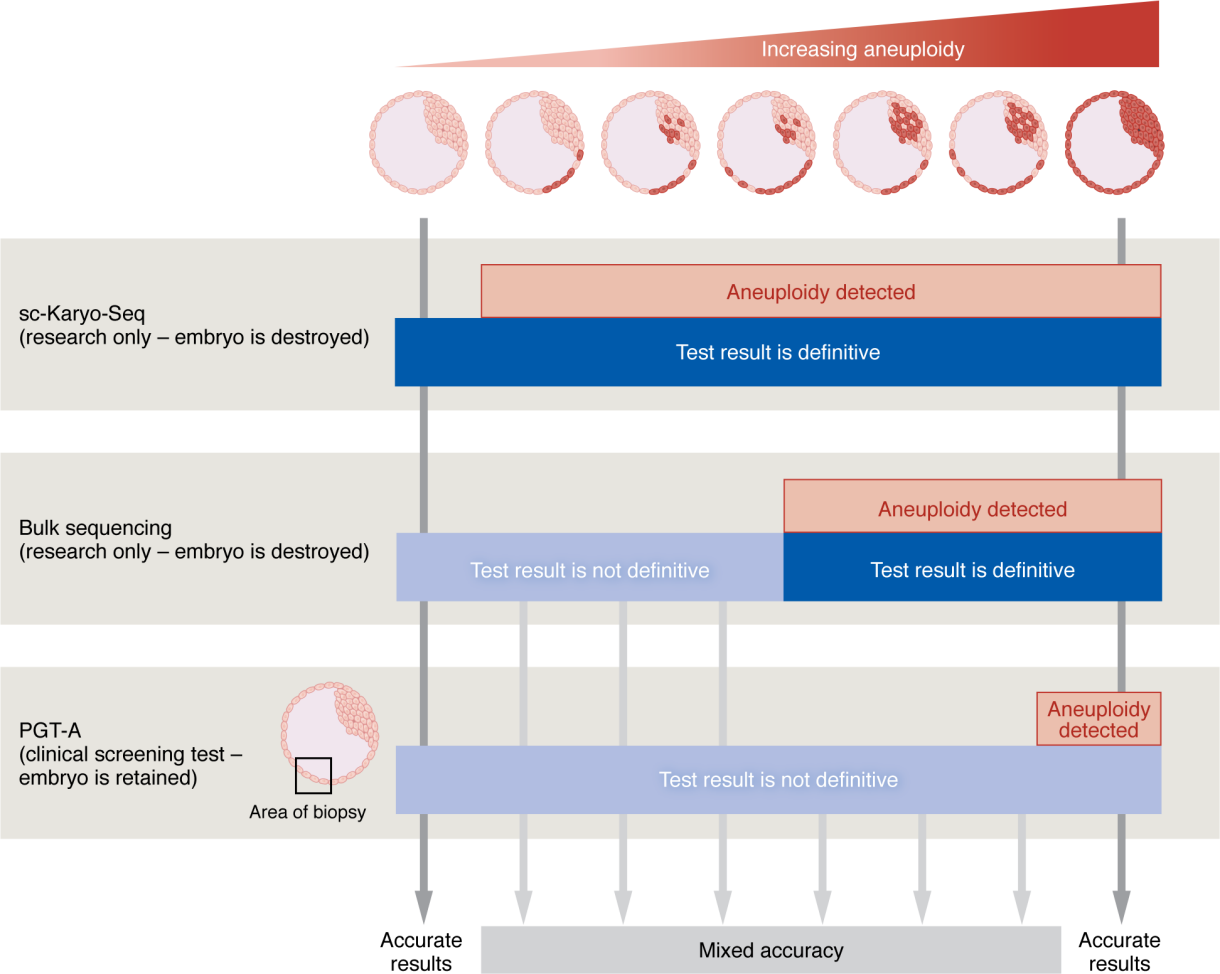
Different genetic analysis platforms have a varying capacity to detect mosaic aneuploidy in blastocyst-stage embryos. Embryos may be euploid (100% normal cells), aneuploid (100% abnormal cells), or mosaic aneuploid, with varying proportions of cells in the inner cell mass and/or trophectoderm affected by numerical and/or structural chromosomal defects. sc-Karyo-Seq analysis of all cells recovered from blastocysts offers high sensitivity and specificity, accurately detecting mosaic aneuploidy in even a small minority of cells. Bulk sequencing techniques analyze entire embryos or embryo parts and have lower sensitivity and specificity than sc-Karyo-Seq, only detecting aneuploidy when at least 20% of the cells in a sample are aneuploid. PGT-A is a clinical test utilizing bulk sequencing to analyze small segments of four to six trophectoderm cells recovered from embryos via biopsy. PGT-A frequently fails to correctly classify aneuploid status in mosaic aneuploid embryos due to low sensitivity of bulk sequencing techniques and spatial heterogeneity of chromosome status, meaning that the segment is not informative of the entire embryo. When none or less than 20% of cells in a segment are aneuploid, the test may return a negative or inconclusive result even when aneuploidy exists. When more than 20% of cells in a segment are aneuploid, the test may return a positive result even if most of the cells in an embryo are normal.

The power of the single-cell sequencing approach means that the chromosome status of each individual cell of entire blastocyst-stage embryos could be analyzed (9). This method contrasts with prior bulk-sequencing techniques that instead sequence pooled cells from whole embryos or segments of embryos. The bulk approach is subject to dilution effects, so that loss or gain of DNA cannot be detected when only 20% or fewer of the cells are abnormal. It also misses some types of anomalies in which reciprocal changes between cells cancel out one another (10, 11). This means that earlier studies using bulk sequencing have routinely undercounted the incidence of chromosomal anomalies and have classified embryos as entirely normal when they may not be (Figure 1).

The Chavli et al. study (9) convincingly demonstrates that embryos with chromosomal abnormalities are far more common than previously thought. They sequenced approximately half of the cells from each of 55 good-quality surplus blastocyst-stage embryos and found that at least 45 of the embryos (82%) contained a proportion of cells with numerical and/or structural DNA faults consistent with mosaic aneuploidy. The majority of these embryos (*n* = 32, 58%) exhibited diploid-aneuploid mosaicism, in which, on average, 60% of cells were normal, indicating a mitotic origin. Defects were similarly common in the trophectoderm layer and inner cell mass compartment of embryos and often affected 20% or fewer of the cells in an embryo — an incidence that is not reliably detectable by bulk DNA-sequencing techniques. A range of chromosomal defects was evident, with similar frequencies of numerical and structural abnormalities. Sequencing results were not obtained for all cells because of technical challenges with cell recovery, and since embryos in which aneuploidy was not found often had lower cell recovery rates, the authors considered it likely that the true rate of mosaic aneuploidy in embryos is even higher than 82%.

The study used good-quality blastocysts made using a high standard of clinical protocols, and the experiments and analysis were technically and scientifically robust. Therefore, we find it compellingly reasonable to expect that mosaicism occurs with similar frequency in all IVF clinics around the world. However, more studies using single-cell approaches will now be necessary to confirm the generalizability of these findings.

## Implications for reproductive medicine are profound

The findings of Chavli et al. have important implications. First, and most crucial, it will be essential to determine whether there are characteristics of patients undergoing IVF or elements of the IVF process that contribute to producing chromosomal mosaicism, or whether mosaicism is a feature of most human embryos regardless of how they are conceived. One study using embryos flushed from the uterus indicates similar rates of aneuploidy in IVF and naturally conceived embryos (12). Given the ethical and technical challenges of sourcing naturally conceived human embryos, studies in nonhuman primates and other animals would be informative on this point. Another worthy study would be to undertake single-cell sequencing in human embryos resulting from standard IVF versus intracytoplasmic sperm injection (ICSI) and to evaluate the effects of embryo freezing and other aspects of the embryology laboratory processes. If all embryos contain mitotic errors regardless of their heritage, it will add confidence to the conclusion that such errors are normal in human embryos.

A major consequence of the Chavli et al. (9) results must be to reconsider the clinical utility of PGT-A. There is no argument against genetic testing for families at high risk of inherited single-gene disease. However, the rationale for routine aneuploidy testing of IVF embryos now seems questionable. PGT-A tests the chromosomal composition of a small cluster of trophectoderm cells from a blastocyst-stage embryo with the expectation of returning a result that enables the selection of embryos free of chromosomal defects. Such testing has been based on assumptions that (a) the biopsied segment is informative of the entire embryo, and (b) finding aneuploidy in the biopsied segment indicates reduced fitness or impaired developmental competence. The Chavli et al. (9) data now firmly counter the validity of these assumptions by proving extensive heterogeneity in the chromosomal status of individual cells in an embryo and demonstrating that the incidence of aneuploidy is too high to be attributable solely to embryos with low developmental potential.

## Caveats and controversies surround PGT-A testing

The Chavli et al. findings align with other recent studies that have raised red flags about PGT-A. Bulk sequencing and microarray approaches have pointed to much higher-than-anticipated rates of chromosomal mosaicism in good-quality IVF embryos and have indicated that segments of the trophectoderm do not reflect the status of the whole embryo (2, 11, 13). Popovic et al. (11) reported that embryos commonly exhibit considerable spatial variation in the position of abnormal cells, such that a segment taken from one site in the trophectoderm layer (that will later form the placenta) can yield an aneuploid result, while similar biopsies from other regions in the trophectoderm layer, or in the inner cell mass that will give rise to the fetus, show normal euploid cells. Previous single-cell sequencing studies of cleavage-stage human embryos have pointed to a high incidence and diverse patterns of chromosomal aberrations (14, 15). Others have shown that the aneuploidy rate declines as development progresses, indicating that many aneuploid cells are selectively lost before embryo implantation (16) and that when the number of affected cells is high, embryos with aneuploid cells often undergo developmental arrest (17).

These data are consistent with several clinical studies showing that inclusion of PGT-A testing in regular clinical practice does not improve the chance of IVF success, known as the take-home-baby rate (18, 19). That embryos with mosaic aneuploidy often result in healthy babies is confirmed (3, 6, 7), although it may be linked with a slightly higher risk of miscarriage in some women (20).

Collectively, these studies strongly indicate that PGT-A testing misclassifies a considerable proportion of embryos and cannot be relied on for definitive results. It seems clear that many embryos will contain abnormal cells despite a negative biopsy test, while others will be classified as faulty and not suitable for transfer, when the majority of cells in the embryo, including the crucial inner cell mass, are normal (Figure 1). The sampling error cannot be overcome by application of single-cell sequencing to PGT-A because this sequencing technique destroys the embryo. Ideally, noninvasive testing approaches that assess the entire embryo and circumvent embryo damage or destruction are required, but to date, no reliable noninvasive approach exists.

## Future priorities and unresolved questions

Given the information provided by Chavli et al. (9), it seems abundantly clear that PGT-A falls short of the technical and ethical standards required of genetic screening tests (21). Careful consideration must now be given to the utility of PGT-A testing with objective assessment of its limitations and risks as well as possible benefits. This is essential for clinicians and patients to make informed choices on accessing the power of genomic testing and to properly consider PGT-A test results. Debates on PGT-A use must be founded in robust and rigorous science, while ensuring that patient care and the health of children are paramount.

The research imperative is to now build on the transformative work of Chavli et al. (9) to better understand the underlying biology of chromosomal mosaicism in embryos — the reasons why it occurs, the usual fate of abnormal cells, and their relevance to ongoing development and infant outcomes. Uneven segregation of chromosomes at mitosis and anomalies in chromosome structure may be elevated after DNA damage — but why aneuploidy should be so prevalent in early embryos is not clear. Clinical data available to date do not point to maternal age as a factor, unlike meiotic errors that increase with age (22). Use of sophisticated imaging approaches implies that the extended prometaphase and metaphase of the first cell division in zygotes contribute to chromosome segregation errors (3). It will be relevant to investigate whether transcriptional inactivity during the first few cell divisions promotes replication stress, such that the replication machinery is more rate limiting in early embryos than other tissues. Another question is how abnormal cells are selectively removed from the developing conceptus. In the physiological setting, immune surveillance might contribute to the removal of abnormal cells (15, 23), but immune cells have limited access to preimplantation embryos compared with later stages of fetal development and other sites in the body.

Given the pivotal importance of events at conception for lifetime health (24), it seems remarkable that such crucial features of early development are so poorly understood. As long as the biology remains uncharted, we are missing the information required to inform best clinical practice and ensure optimal outcomes for children and families.

## Version 1 03/15/2024

Electronic publication
